# Effects of Bilateral Subthalamic Nucleus Stimulation on Depressive Symptoms and Cerebral Glucose Metabolism in Parkinson’s Disease: A ^18^F-Fluorodeoxyglucose Positron Emission Tomography/Computerized Tomography Study

**DOI:** 10.3389/fnins.2022.843667

**Published:** 2022-06-01

**Authors:** Xiaoxiao Zhang, Huiwei Zhang, Zhengyu Lin, Daniel A. N. Barbosa, Yijie Lai, Casey H. Halpern, Valerie Voon, Dianyou Li, Chencheng Zhang, Bomin Sun

**Affiliations:** ^1^Department of Neurosurgery, Ruijin Hospital, School of Medicine, Shanghai Jiao Tong University, Shanghai, China; ^2^PET Center, Huashan Hospital, Fudan University, Shanghai, China; ^3^Department of Neurosurgery, School of Medicine, Stanford University, Stanford, CA, United States; ^4^Department of Psychiatry, School of Clinical Medicine, University of Cambridge, Cambridge, United Kingdom

**Keywords:** brain metabolism, Parkinson’s disease, positron emission tomography, subthalamic nucleus deep brain stimulation, treatment-resistant depression

## Abstract

Subthalamic nucleus (STN) deep brain stimulation (DBS) can improve motor symptoms in Parkinson’s disease (PD), as well as potentially improving otherwise intractable comorbid depressive symptoms. To address the latter issue, we evaluated the severity of depressive symptoms along with the severity of motor symptoms in 18 PD patients (mean age, 58.4 ± 5.4 years; 9 males, 9 females; mean PD duration, 9.4 ± 4.4 years) with treatment-resistant depression (TRD) before and after approximately 1 year of STN-DBS treatment. Moreover, to gain more insight into the brain mechanism mediating the therapeutic action of STN-DBS, we utilized ^18^F-fluorodeoxyglucose (FDG) positron emission tomography (PET) to assess cerebral regional glucose metabolism in the patients at baseline and 1-year follow-up. Additionally, the baseline PET data from patients were compared with PET data from an age- and sex-matched control group of 16 healthy volunteers. Among them, 12 PD patients underwent post-operative follow-up PET scans. Results showed that the severity of both motor and depressive symptoms in patients with PD-TRD was reduced significantly at 1-year follow-up. Also, patients used significantly less antiparkinsonian medications and antidepressants at 1-year follow-up, as well as experiencing improved daily functioning and a better quality of life. Moreover, relative to the PET data from healthy controls, PD-TRD patients displayed widespread abnormalities in cerebral regional glucose metabolism before STN-DBS treatment, which were partially recovered at 1-year follow-up. Additionally, significant correlations were observed between the patients’ improvements in depressive symptoms following STN-DBS and post-operative changes in glucose metabolism in brain regions implicated in emotion regulation. These results support the view that STN-DBS provides a promising treatment option for managing both motor and depressive symptoms in patients who suffer from PD with TRD. However, the results should be interpreted with caution due to the observational nature of the study, small sample size, and relatively short follow-up.

## Introduction

Parkinson’s disease (PD) is the second most common neurodegenerative disorder, being composed of disabling motor and non-motor symptoms (Armstrong and Okun, 2020). The latter include hyposmia, sleep disorders, constipation, and mood disturbances (Schapira et al., 2017). About 35% of patients with PD experience clinically relevant depressive symptoms (Aarsland et al., 2011). Depression in patients with PD forms a key determinant of poor health-related quality of life and is associated with reduced cognitive functioning (Aarsland et al., 2011). Unfortunately, depression can be persistent in some PD patients, even after treatment with established conventional pharmacological and psychological therapies.

The subthalamic nucleus (STN), which serves as a crucial relay node in cortical-basal ganglia motor networks (Hamani et al., 2017), is a well-established target in deep brain stimulation (DBS) treatment for advanced PD (Limousin and Foltynie, 2019). Moreover, the STN is functionally interconnected with the prefrontal cortex, as well as with the limbic and association sub-regions of the basal ganglia, which are involved in mood, motivation, and cognitive functions (Lhommée et al., 2012). Accordingly, it has been hypothesized that STN-DBS could modulate not only the motor symptoms but also the non-motor symptoms of PD, including depressive symptoms (Fasano et al., 2010; Combs et al., 2015). Indeed, some studies have reported a beneficial effect of STN-DBS on depressive symptoms in PD (Witt et al., 2008; Castrioto et al., 2014; Combs et al., 2015). However, the efficacy of STN-DBS for severe and medically intractable depression, or treatment-resistant depression (TRD), has not yet been well evaluated in patients with PD. Furthermore, not much is known regarding the neurobiological mechanism underpinning the hypothesized antidepressant effect of STN-DBS in PD.

A positron emission tomography (PET) study reported that STN-DBS increased glucose hypometabolism in the associative and limbic areas of the basal ganglia and various cortical areas, including the frontal cortex, as well as suppressing cerebellar hypermetabolism in PD (Hilker et al., 2004), some regions of which may also be associated to pathogenesis of depression (Su et al., 2014). Another PET study indicated that STN-DBS alleviated depressive symptoms in PD by decreasing glucose hypermetabolism in thalamic and striatal structures (e.g., right thalamus, left caudate, right lateral globus pallidus) and by increasing glucose hypometabolism in cortical areas (e.g., left superior and middle temporal cortex, right precuneus, inferior parietal lobule) (Smith et al., 2019). However, the results of these studies should be considered cautiously due to small sample size and lack of control group.

In the present prospective study, we aimed to gain a better understanding of the clinical effectiveness of STN-DBS for treating PD patients with TRD (PD-TRD), as well as gaining insight into the neurobiological mechanisms that could be responsible for its therapeutic action. To this end, we utilized ^18^F-FDG-PET computerized tomography (CT) to evaluate motor symptoms, depressive symptoms, and cerebral regional glucose metabolism in 18 PD-TRD patients before and after they received approximately 1 year of STN-DBS treatment. Moreover, we compared the PET data from patients at baseline with PET data collected from a control group of age- and sex-matched healthy volunteers. Additionally, we explored whether the potential antidepressant action of STN-DBS treatment at 1-year follow-up would be paralleled by post-operative changes in the PET-based measures of cerebral regional glucose metabolism.

## Materials and Methods

### Participants

Eighteen patients with PD-TRD and 16 healthy volunteers were enrolled in this study. Both patients and healthy controls were recruited from the Center for Functional Neurosurgery at Ruijin Hospital (Shanghai, China). Because 6 patients declined to participate in post-operative PET imaging session, 12 patients were left for analyzing post-operative changes in cerebral regional glucose metabolism. At the time of STN-DBS surgery, diagnosis was made by a neurologist specialized in movement disorders and two independent psychiatrists using DSM-IV criteria (American Psychiatric Association, 2013). TRD was defined as a failure to respond to: (1) At least two different classes of antidepressants with adequate dose and treatment duration (including augmentation or combination strategies with lithium, atypical antipsychotics, or anticonvulsants), and (2) Evidence-based psychotherapy (McIntyre et al., 2014). In addition, the patients had to meet several STN-DBS surgical inclusion criteria, as described elsewhere (Li et al., 2017). Given the purpose of this study, the presence of severe depression was not a contraindication for DBS surgery. Patients were under close supervision by a multidisciplinary medical team before and after STN-DBS surgery to manage possible changes in clinical state, including suicide attempts. No significant adverse events, including deaths, occurred throughout the study. The healthy control group consisted of 16 healthy volunteers who were matched to patients by age and sex (Chi-square test for gender comparison between the two groups), and had no previous or current metabolic, neurological, or psychiatric disorders. The study was approved by the ethics committee of Ruijin Hospital, Shanghai Jiao Tong University School of Medicine. Written informed consent was obtained from each participant before study participation.

### Surgical Procedures and Programming

Surgeries were performed in the Center for Functional Neurosurgery at Ruijin Hospital. Under local anesthesia and light sedation, quadripolar electrodes (Model 3387; Medtronic, Minneapolis, MN, United States) were implanted bilaterally using the Leksell stereotactic frame (Elekta, Stockholm, Sweden), guided by co-registered high-resolution magnetic resonance imaging (MRI) (3 Tesla, General Electric, Madison, WI, United States) and CT. We obtained post-operative CT from all patients to verify the accuracy of electrode placement. Programming was conducted using standardized protocols (Picillo et al., 2016; Chen et al., 2018), and was initiated 1 week after surgery. The stimulation contacts were chosen based on the location of the implanted electrodes and the patient’s acute clinical response, as well as on the emergence of side effects to the stimulation delivered. In most cases, the DBS parameters were optimized 3 months after surgery. The mean amplitude, pulse width, and frequency delivered to the right side of the STN were 2.0 ± 0.2 V, 73.3 ± 14.0 μs, and 126.7 ± 8.2 Hz. The clinically most optimal parameter values for the left STN were 2.1 ± 0.2 V, 78.0 ± 13.7 μs, and 125.3 ± 6.4 Hz.

### Clinical Outcome Assessment

Clinical assessments of patients were conducted before STN-DBS surgery (baseline) and at 1-year follow-up. The Unified Parkinso’s Disease Rating Scale-Motor Part (UPDRS-III) was utilized to evaluate the severity of motor symptoms (Fahn, 1987). The levodopa equivalent daily dose (LEDD) was calculated according to Tomlinson et al. (2010). The severity of depressive symptoms was assessed by the 24-item Hamilton Depression Rating Scale (HAMD-24). In addition, the Activity of Daily Living (ADL) and 8-item Parkinson’s Disease Questionnaire (PDQ-8) were used to assess the quality of patients’ daily living activities and health-related quality of life. We also utilized the Mini Mental State Examination (MMSE) to assess global cognitive functioning and to monitor possible cognitive changes following STN-DBS surgery. For each clinical assessment instrument, we employed a standardized shorter Chinese version with satisfactory validity and reliability. Finally, we obtained HAMD-24, ADL, and MMSE data from the healthy control participants.

### Positron Emission Tomography Imaging and Preprocessing

Patients participated in two PET imaging sessions, one taking place before STN-DBS surgery and the other at 1-year follow-up, with a pre-imaging withdrawal period for antiparkinsonian and antidepressant medications of at least 12 h. Because patients found it hard to undergo the PET procedure under the off-medication/off-stimulation condition, we performed PET imaging only under the off-medication/on-stimulation condition. Additionally, healthy volunteers participated in one PET imaging session. Both patients and healthy volunteers were instructed not to eat for at least 6 h beforehand. All individual serum glucose levels were measured and fell within the normal range before PET imaging. Participants were first administered intravenously a 185 MBq injection of ^18^F-FDG under standardized conditions (e.g., taking place in a quiet, dimly lit medical room, using a head-holder to immobilize the participant’s head, instructing the participant to keep the eyes open). Forty-five minutes after having received the intravenous injection of ^18^F-FDG, a 10-min three-dimensional (3-D) emission scan was acquired using the Biograph 64 HD PET/CT system (Siemens, Munich, Germany). Attenuation correction was performed based on the CT scan (150 mAs, 120 kV, Acq. 64.0 × 0.6 mm) acquired before PET imaging. After correcting for scatter, dead time, and random coincidence detection, the PET images were reconstructed using a 3-D filtered back-projection and Gaussian filter [full-width half-maximum (FWHM), 3.5 mm], which provided 64 continuous slices in the transversal plane with 5-mm spacing.

The neuroimaging data were preprocessed using SPM5 (Statistical Parametric Mapping Software, version 5; Wellcome Department of Imaging Neuroscience, Institute of Neurology, London, United Kingdom) implemented in MATLAB 2009b (MathWorks, Inc., Sherborn, MA, United States). Montreal Neurological Institute (MNI) templates were used for appropriate linear and non-linear 3-D transformations, which were then used for single-subject spatial normalization. Subsequently, the PET images were smoothed by using a Gaussian filter of 10 mm FWHM over a 3-D space to improve the signal-to-noise ratio. The PET data were then subjected to statistical analysis.

### Statistical Analysis

#### Clinical Data

We used paired-samples *t*-test to evaluate whether the patients’ scores on the UPDRS-III, HAMD-24, ADL, PDQ-8, and MMSE observed before STN-DBS treatment differed significantly from the scores obtained at 1-year follow-up. Independent-samples *t*-tests were utilized to assess patient-control differences at baseline. Also, we conducted a correlation analysis and linear regression on the data from patients to assess the relationship between post-operative changes in the HAMD-24 score, as well as in the UPDRS-III score, and post-operative changes in the PET-based measures of regional cerebral glucose metabolism. The Statistical Package for the Social Sciences (SPSS, version 22; IBM, Chicago, IL, United States) was used to analyze the data, adopting a *p*-value < 0.05 (two-tailed) as being significant. Data are presented as mean with standard deviation (SD), unless otherwise indicated. We also present a treatment effect size estimate, namely, Hedges’ *g*, using the average standard deviation of both repeated measures as a standardizer (Lakens, 2013). Hedges’ *g* is similar to Cohen’s *d* but is better suited when sample size is small. Hedges’ *g* values of 0.2, 0.5, and 0.8 are typically considered as a small, medium, and large effect, respectively.

#### Positron Emission Tomography Data

To assess whether patients and healthy control participants differed from each other in whole-brain glucose metabolism rates, an independent-samples *t*-test was conducted by means of a whole-brain voxel-wise analysis in SPM5. Overall group differences were removed by using proportional scaling, with the global mean set to 50 and the threshold masking to 0.8. Clusters of at least 150 voxels were considered as significantly different (i.e., either hypermetabolism or hypometabolism) using an uncorrected threshold (*p* < 0.001, two-tailed). For all statistical analyses, the Talairach client was used to convert the MNI coordinates into Talairach coordinates. Furthermore, we assessed patient-control group differences in cerebral regional glucose metabolism using a volume of interest (VOI) analysis. To quantify metabolic rates in specific brain regions, we used a 4-mm radius spherical VOI centered around significant clusters with a peak-level extent thresholding based on the certain coordinates from SPM results. These certain coordinates represent the brain regions with the most significant differences between groups. We then used Scan/VP software (version 5.9.1; Center for Neuroscience, Feinstein Institute for Medical Research, Manhasset, NY, United States) to calculate relative cerebral glucose metabolism values (i.e., globally adjusted) for patients (18 patients before and 12 patients after STN-DBS treatment) and 16 healthy volunteers.

## Results

### Sample Characteristics

Table 1 summarizes the demographic and clinical data obtained from the 18 patients with PD-TRD and 16 healthy controls enrolled in the study, along with the data from only those patients (*n* = 12) who participated in both the pre-operative and post-operative PET imaging sessions. Six patients participated in the pre-operative PET imaging session but declined to participate in the PET session at 1-year follow-up due to a medical reason (orthopedic fracture, *n* = 1) or unwillingness to undergo the PET procedure again (*n* = 5). There were no significant differences between the patients who either did or did not undergo PET imaging at follow-up in any of the demographic and clinical variables assessed (age, *p* = 0.384; sex ratio, *p* = 1.000; UPDRS-III, *p* = 0.435; HAMD-24, *p* = 0.111; MMSE, *p* = 0.359). Also, the clinical benefits from STN-DBS at 1-year follow-up, as described in the next section, were similar regardless of whether the 6 patients who declined to undergo post-operative PET imaging were included or excluded from analysis (Table 2).

**TABLE 1:** Demographics of HC and PD-TRD patients at baseline.

Items	HC (*n* = 16)	PD-TRD patients (*n* = 18)	PD-TRD patients (*n* = 12)*^a^*
Sex	10M/6F	9M/9F	6M/6F
Age	54.8 ± 7.3*	58.4 ± 5.4	59.3 ± 4.4**
Duration of PD	/	9.4 ± 4.4	8.4 ± 4.8
UPDRS-III	/	39.2 ± 7.2	38.3 ± 5.9**
HAMD	2.6 ± 2.0	31.7 ± 7.0	33.6 ± 4.9**
MMSE	28.9 ± 1.5	24.4 ± 3.0	23.9 ± 3.4**
ADL	100	36.9 ± 14.1	35.4 ± 15.3**
PDQ-8	/	25.9 ± 2.8	27.1 ± 1.7**
Levodopa equivalent dose (mg/d)	/	619.4 ± 251.6	587.5 ± 222.7
Antidepressant therapies at the time of admission (No. of patients)	/	One class of antidepressant (*n* = 13)	One class of antidepressant (*n* = 8)
		Two classes of antidepressants and/or antipsychotics (*n* = 6)	Two classes of antidepressants and/or antipsychotics (*n* = 4)

*Plus–minus values are mean ± SD.*

^a^PD-TRD patients (n = 12) were defined as 12 of the 18 patients enrolled who underwent PET scan after surgery. *The two groups (HC group and 18 PD-TRD patients) were tested by independent sample T test, and the P value of age was greater than 0.05. **There was no significant difference in age, UPDRS-III, HAMD, MMSE, ADL, and PDQ-8 between PD-TRD patients (n = 18) and PD-TRD patients (n = 12) with post-operative pet (P > 0.05). PD-TRD, Parkinson’s disease with treatment-resistant depression.

**TABLE 2:** Clinical measurements before and after STN-DBS for PD-TRD patients.

	Baseline PD-TRD (*n* = 18)	Follow-up^$^ PD-TRD (*n* = 18)	*P* value	Baseline PD-TRD (*n* = 12)*	Follow-up^$^ PD-TRD (*n* = 12)*	*P* value
^#^UPDRS-III	39.2 ± 7.2	18.1 ± 4.2	1.1 × 10-13	38.3 ± 5.9	18.3 ± 4.0	1.8 × 10^−10^
HAMD-24	31.7 ± 7.0	7.4 ± 2.7	4.8 × 10-12	33.6 ± 4.9	7.3 ± 2.6	4.5 × 10^−10^
MMSE	24.4 ± 3.0	25.4 ± 2.9	0.095	23.9 ± 3.4	25.3 ± 2.9	0.1
ADL	36.9 ± 14.1	88.1 ± 14.9	2.1 × 10-10	35.4 ± 15.3	85.4 ± 17.6	1.0 × 10^−16^
PDQ-8	25.9 ± 2.8	6.9 ± 3.5	7.2 × 10-14	27.1 ± 1.7	7.8 ± 4.0	6.8 × 10^−9^
Levodopa equivalent dose (mg/d)	619.4 ± 251.6	341.7 ± 157.4	0.037	587.5 ± 222.7	345.8 ± 187.6	3.8 × 10^−4^

Plus–minus values are mean ± SD. ^$^The mean follow-up duration was 14.7 ± 3.1 months. * PD-TRD patients (n = 12) were 12 of the 18 patients enrolled who underwent PET scan after surgery. ^#^UPDRS-III was evaluated in off-medication condition. STN-DBS, subthalamic nucleus deep brain stimulation; PD-TRD, Parkinson’s disease with treatment-resistant depression; UPDRS-III, Unified Parkinson’s Disease Rating Scale-Motor Part; HAMD-24, 24-item Hamilton Depression Rating Scale; ADL, Activity Daily Living Scale; MMSE, Mini-Mental State Examination; PDQ-8, 8-item Parkinson’s Disease Questionnaire.

In addition, statistical analysis showed that the patients at baseline did not differ significantly from the healthy control participants in terms of age and sex ratio (*p* = 0.114 for age. Chi-square test was performed for the gender of HC group and PD group, *p* = 0.464), while showing a significantly higher level of depressive symptoms, impaired daily living activities, and a lower overall level of cognitive functioning (Table 1). Also, all patients were taking antiparkinsonian and antidepressant medications. The mean duration of pre-operative antidepressant medication therapy was 17.8 ± 8.3 weeks.

### Clinical Data

At 1-year follow-up, the severity of patients’ motor symptoms was reduced by 54.0 ± 5.6% compared with the severity of their symptoms observed at baseline (Hedges’ *g* = 0.34 for UPDRS-III score difference in the off-medication state), along with showing a significant reduction in LEDD (Table 2). Moreover, the severity of depressive symptoms, as indexed by the HAMD-24 score, was reduced significantly at 1-year follow-up (Hedges’ *g* = 0.44). Similarly, 6 patients no longer took any antidepressant or antipsychotic medications at 1-year follow-up, while the remaining 12 patients were taking only 1 class of antidepressant medication with substantial dose reduction.

At 1-year follow-up, the patients also experienced significant improvements in their daily living activities (Hedges’ *g* = 0.34 for ADL score difference) and health-related quality of life (Hedges’ *g* = 0.57 for PDQ-8 score difference). No significant differences in global cognitive function, as assessed by the MMSE, were seen following 1 year of STN-DBS treatment, The HAMD scores of both pre-operative and post-operative PD groups were in line with normal distribution (Table 2).

### Positron Emission Tomography Data

#### Patient-Control Differences

**FIGURE 1:**
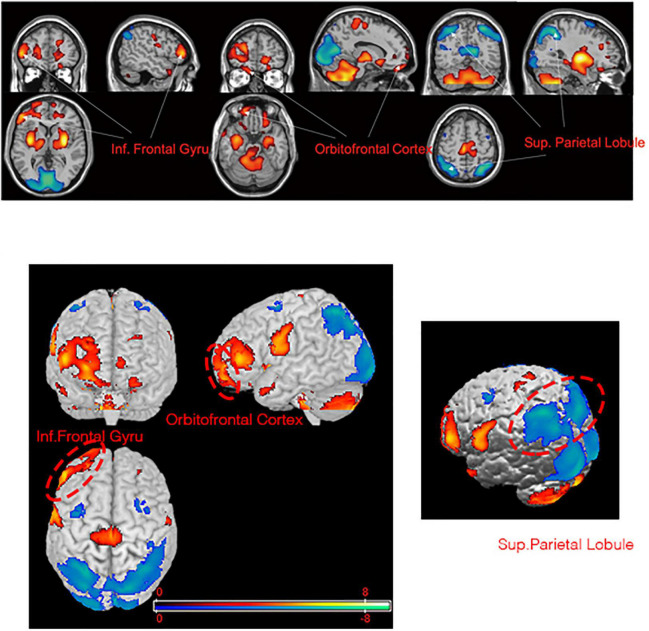
Brain regions with significant metabolic differences in PD-TRD patients versus normal individuals (i.e., the health controls). Glucose metabolism in PD-TRD patients is increased (red) in the right OFC and bilateral inferior frontal gyrus and is decreased (blue) bilaterally in the superior parietal gyrus.

**FIGURE 2:**
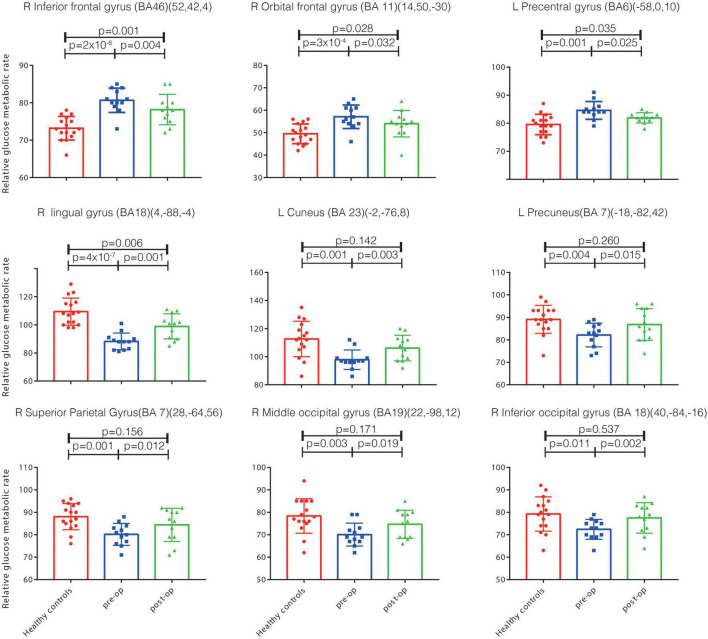
The relative glucose metabolism rates in the health controls and in patients pre-op and post-op. Significant metabolism differences exist between the health controls and pre-op PD-TRD patients (*p* < 0.05), and between the pre-op and post-op patients (*p* < 0.05). However, no significant metabolism differences exist between the post-op patients and health controls in the left cuneus, precuneus, right inferior occipital gyrus, middle occipital gyrus, superior parietal gyrus (*p* > 0.05). Inf., inferior; Sup., superior; BA, Brodmann’s area; post-op, post-operation; pre-op, pre-operation; PD, Parkinson’s disease; DBS, deep brain stimulation; PD-TRD, Parkinson’s disease with treatment-resistant depression; OFC, orbitofrontal cortex.

We observed significant differences between patients at baseline and healthy control participants in the PET-based measures of glucose metabolism in various cortical and subcortical regions (Figures 1, 2 and Supplementary Table 1). Specifically, patients were characterized by a relative hypermetabolism in the bilateral inferior frontal gyrus [Brodmann’s area (BA) 46], bilateral anterior cingulate (BA32), and the right orbitofrontal cortex (OFC; BA11). By contrast, a relative hypometabolism was found in the bilateral lingual gyrus (BA18), bilateral cuneus (BA23), bilateral superior parietal lobule (BA7), bilateral middle occipital gyrus (BA19), bilateral inferior parietal lobule (BA40), and left middle temporal gyrus (BA21).

#### Differences Between Baseline and Follow-Up

The patients’ cerebral regional glucose metabolism at baseline was markedly changed after 1 year of STN-DBS treatment (Figure 2). Specifically, the patients showed significantly decreased glucose metabolism rates in the right OFC (BA11), right inferior frontal gyrus (BA46) and left precentral gyrus (BA6) at 1-year follow-up relative to baseline levels. Similarly, significantly increased glucose metabolism rates were observed in the right lingual gyrus (BA18), right superior parietal gyrus (BA7), right middle occipital gyrus (BA19), right inferior occipital lobule (BA18), left precuneus (BA7), and left cuneus (BA23) (Figure 2).

Furthermore, a comparison of the follow-up data with the PET data from healthy control participants indicated that the patients’ glucose metabolism rates in the left cuneus, left precuneus, right superior parietal gyrus, right middle occipital gyrus, and right inferior occipital gyrus were changed at 1-year follow-up, reaching values that were not significantly different from those of healthy controls (*p* > 0.05, Figure 2). These regions all survived false discovery rate correction (*p* ≤ 0.001).

#### Correlations Between Clinical and Positron Emission Tomography Outcome Data

**FIGURE 3:**
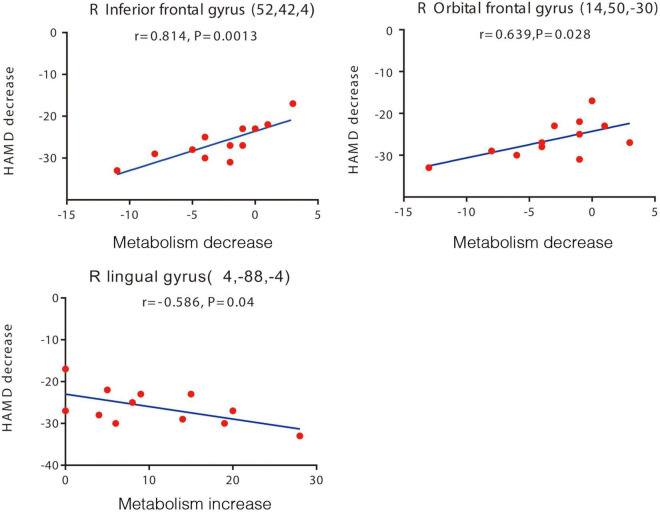
Brain regions with significant correlations between changes in the HAMD-24 score and changes in glucose metabolism after STN-DBS. Decreased HAMD-24 scores are correlated with decreased metabolism in the right inferior frontal gyrus, right orbital frontal gyrus (upper two panels), and with increased metabolism in the right lingual gyrus post-operation (lower panel). Each volume of interest peak coordinates is in parentheses in each panel. The threshold of the SPM5 map (Wellcome Department of Imaging Neuroscience, Institute of Neurology, London, United Kingdom) is *t* = 3.14 (*p* < 0.01). The map is superimposed on a standard magnetic resonance imaging brain template. The y-axis in this figure is the pre-operative and post-operative difference of HAMD score, and the x-axis is the pre-operative and post-operative difference of relative metabolic rate. HAMD-24, 24-item Hamilton Depression Rating Scale; STN-DBS, subthalamic nucleus deep brain stimulation.

The patients’ reductions in the HAMD-24 score observed at 1-year follow-up correlated significantly with post-operative changes in the PET-based measures of cerebral regional glucose metabolism. Particularly, post-operative HAMD-24 score reductions showed positive correlations and linear relationships with post-operative reductions in glucose metabolism in the right inferior frontal gyrus (*r* = 0.814, *p* = 0.001) and right OFC (*r* = 0.639, *p* = 0.028) (Figure 3). Post-operative reductions in glucose metabolism in these two regions also showed positive correlations with post-operative reductions in the UPDRS-III score (right inferior frontal gyrus: *r* = 0.609, *p* = 0.036; right OFC: *r* = 0.715, *p* = 0.009). Thus, relatively large post-operative score reductions on the HAMD-24 or UPDRS-III were associated with relatively large post-operative reductions in glucose metabolism in the right inferior frontal gyrus and right OFC. Additionally, a significant negative correlation and linear relationship was detected between post-operative reductions in the HAMD-24 score and post-operative reductions in glucose metabolism in the right lingual gyrus (*r* = -0.586, *p* = 0.040) (Figure 3).

There were no significant correlations detected between the post-operative reductions in the HAMD-24 score and post-operative changes in the scores on the UPDRS-III (off-medication: *r* = 0.19, *p* = 0.45), ADL (*r* = 0.17, *p* = 0.50), MMSE (*r* = 0.11, *p* = 0.76), and PDQ-8 (*r* = 0.35, *p* = 0.13).

Beyond that, there were no significant correlations detected between the post-operative reduction in MMSE and FDG uptake. We correlated the post-operative glucose metabolic difference of 9 VOI (right inferior frontal gyrus, right orbital frontal gyrus, left precentral gyrus, right lingual gyrus, left cuneus, left precuneus, right superior parietal gyrus, right occipital gyrus, right inferior occipital gyrus) with the pre-operative and post-operative MMSE difference of patients, the *p* values were greater than 0.05 (*p* = 0.235, *p* = 0.165, *p* = 0.726, *p* = 0.738, *p* = 0.788, *p* = 0.590, *p* = 0.208, *p* = 0.102, *p* = 0.531, respectively).

## Discussion

In this study, we observed that STN-DBS treatment was associated with significant improvements in both motor and depressive symptoms in patients who suffer from PD with TRD, with small-to-moderate treatment effects (Hedges’ *g* = 0.34 and 0.44, respectively). Moreover, the patients’ intake of antiparkinsonian and antidepressant medications was substantially reduced at 1-year follow-up. They also experienced significant improvements in performing daily living activities and in their health-related quality of life. There were no changes detected in the patients’ cognitive status following 1 year of STN-DBS. Hoops et al. (2009) suggested that the optimal diagnostic cutoff point of PD for the MMSE with mild cognitive impairment was 23/24. The pre-operative and post-operative MMSE scores of enrolled patients were higher than this cutoff point. Even so, mild cognitive impairment is not an absolute contraindication. In our research, no significant adverse side effects occurred during the follow-up period. These results not only confirm the established efficacy of STN-DBS for motor symptoms in PD, but also substantiate findings of previous studies (Witt et al., 2008; Castrioto et al., 2014; Combs et al., 2015), indicating that STN-DBS may also have an antidepressant action in patients with PD-TRD.

In addition, we found that the patients with PD-TRD displayed widespread abnormalities in the PET-based measures of cerebral regional glucose metabolism when compared with PET data collected from age- and sex-matched healthy control participants. The abnormalities took the form of a relative hypermetabolism of glucose in frontal brain regions, particularly the inferior frontal gyrus and right OFC, along with a relative hypometabolism in posterior regions, including the inferior and superior parietal lobule, lingual gyrus, middle occipital gyrus, and left middle temporal gyrus (Supplementary Table 1). Moreover, the patients’ abnormalities of glucose metabolism in some brain areas affected were recovered after 1 year of STN-DBS treatment, reaching levels not significantly different than those seen in healthy control participants (Figure 2). These results suggest that STN-DBS may have improved a variety of brain functions in patients, including motor control and emotion regulation.

### The Orbital Frontal Cortex

The abnormal glucose metabolism rates observed in regions of the frontal lobe in PD-TRD patients at baseline and partial restorations in these brain regions after STN-DBS seem to be particularly relevant to the pathophysiology and management of depressive symptoms in PD-TRD patients. The OFC is considered a core element of widely distributed neural network, which includes the amygdala, ventral stratum, insula, and cingulate cortex, involved in motivation, learning, and emotion (Kringelbach and Rolls, 2004; Rolls and Grabenhorst, 2008), and in the pathophysiology of depressive symptoms in PD-TRD in particular. Notably, Rao et al. (2018) reported that unilateral stimulation of the lateral OFC produced acute and dose-dependent improvement in the mood of patients with moderate-to-severe depression. A decreasing cerebral glucose metabolism in OFC has also been observed in TRD patients receiving subgenual cingulate DBS (Mayberg et al., 2005). Consistently, we observed that the improvement in depressive symptoms after STN-DBS was associated with a partial recovery of glucose hypermetabolism in the patients’ right OFC.

### The Inferior Frontal Gyrus

Furthermore, the glucose hypermetabolism of the right inferior frontal gyrus was partially decreased and the decreased rate was significantly correlated with depressive symptoms improvement after STN-DBS. Parkinson’s disease related pattern (PDRP) (Huang et al., 2007; Wu et al., 2013; Tomše et al., 2017) is a unique glucose metabolism pattern in PD which mainly related to the motor symptom of PD and significantly correlated with UPDRS-III. The pattern was characterized by increases in pallidothalamic, pontine, and cerebellar metabolic activity associated with relative reductions in the lateral premotor cortex, supplementary motor area, and posterior association cortices (Ma et al., 2007). However, the inferior frontal gyrus was not included in this pattern. It suggested that though the inferior frontal gyrus were considered correlated with change in UPDRS-III score, it was not the mainly affect the motor function. On the other hand, the right inferior frontal gyrus was reported to be functionally connected to the OFC (Rolls et al., 2020), making it difficult to attribute the patients’ motor and mood outcomes to altered glucose metabolism in only a single brain area. And its disruption of inferior frontal gyrus could contribute to the altered cognition and attentional bias toward negative emotional stimuli commonly found among patients with depression (Tao et al., 2013).

The inferior frontal gyrus exhibited hyperactivity to working memory tasks in depressed patients compared to the control group (Harvey et al., 2005). Ten individuals meeting DSM-IV criteria for Major Depression and 10 healthy controls were tested with a verbal version of the n-back task during functional MRI (fMRI) scanning. The study found that the working memory network of patients with depression showed hyperactivity when performing n-back tasks. Hyperactive brain regions include the inferior frontal gyrus. Depression is likely the result of maladaptive functional interactions among a network of limbic–cortical regions (Mayberg, 1997).

### The Parietal Cortex

The result to the glucose hypometabolism detected in the parietal lobe (BA7 and BA40) (Supplementary Table 1 and Figure 2) of patients before STN-DBS and to the partial restoration of their metabolic rates in these regions post-surgery (Figure 2), given the established role of the parietal cortex in visual attention and information processing (Bisley and Goldberg, 2010). Indeed, a meta-analytic review of voxel-based morphometric studies indicated that patients with depression often had gray-matter volume abnormalities of the parietal cortex, along with volumetric abnormalities of the prefrontal cortex (Su et al., 2014).

The OFC clusters were connected to other regions in parietal cortices, temporal, and subcortical areas in the striatum and the midbrain. These unique patterns may contribute to the specific functional roles of OFC in depressed PD patients for reward processing, learning, and decision making (Kahnt et al., 2012). In our study, the improvement of patients’ depressive symptoms after operation is related to the metabolic changes of OFC, and the parietal lobe is connected with OFC (Kahnt et al., 2012). Both of them seems to be in the same metabolic network and have sequential changes.

### The Lingual Gyrus

Our finding that STN-DBS partially recovered glucose metabolism increased in the lingual gyrus and that the restoration rate was associated with the improvement in depressive symptom is consistent with the results of a previous PET imaging study (Germain et al., 2007). The latter reported that patients with depression exhibited increased perfusion in the lingual cortices during the evening when compared to the morning, which was accompanied by substantial improvement in mood.

The lingual gyrus and occipital lobe have been reported to be involved in the visual recognition and are believed to play a role in episodic memory consolidation. Kong et al. (2015) reported that happiness and loneliness are associated with activity of the lingual gyrus, which suggested the lingual gyrus is involved in emotional processes. A few studies have demonstrated that depression patients have abnormal changes in the lingual gyrus and occipital lobe. Yang et al. (2015) found that depression patients have significant gray matter volume decreases in the lingual gyrus.

Our study suggested that PD patients with treatment-resistant depression may benefit from STN-DBS and that STN-DBS may lead to improvements in depression symptom in a 1-year follow-up. However, there was no significant correlation between the change in the HAMD scores and the change in UPDRSIII scores. Previous drug therapy for PD indicated that improvement of depressive symptom is not related to the improvement of motor symptom (Burn, 2002).

After STN-DBS, the motor symptoms of patients were significantly improved, the equivalent dose of levodopa could be significantly reduced, and the quality of life was significantly Improved (Zhou et al., 2019). However, the improvement of depression is not only due to the improvement of motor symptoms, but also related to the course of PD and the position of STN in the cortical-basal-ganglia-thalamus-cortical circuit.

Studies suggest that in the early stage of PD, degeneration of relevant nuclei of pons resulting in autonomic nervous dysfunction of patients (Braak et al., 2004; Kostić et al., 2010). In the middle and late stage of PD, the limbic system is involved, the cingulate gyrus and orbitofrontal gyrus become thinner, and patients suffer from mental symptoms such as depression, hallucinations, and cognitive impairment.

The STN is located at the intersection of limbic and cortico-striato-thalamo-cotical networks (Feldmann et al., 2008; Cardoso et al., 2009), which have specific motor, sensorimotor, and limbic functions, and is an important part of the limbic system (Petersson et al., 2019). The circuits form the elementary pathways through which regions of the brain communicate with the basal ganglia (Temel et al., 2005). Each of these circuits originates from specific parts of the cortex, are processed in specific thalamic nuclei and project back to at least one of the cortical input areas. The STN receives and projects to a number of different regions inside and outside the basal ganglia (Hamani et al., 2004).

One study found that Parkinson’s patients had different emotional responses to facial recognition before and after STN-DBS surgery (Le Jeune et al., 2008). A group of 13 patients with Parkinson’s disease underwent ^18^F-FDG PET and recognition of facial emotions (RFE) 3 months before and 3 months after STN DBS surgery. The researchers observed a significant reduction in fear perception after surgery, and there was a positive correlation between these neuropsychological assessments and changes in glucose metabolism, especially in the right OFC. The results of this study confirm the role of STN as a key basal ganglia structure in associative and limbic circuitry.

A PET study reports that, in line with the observations of limbic and associative dysfunction in patients following STN-DBS, the metabolic data, obtained with a large series of PD patients, confirm the central role played by the STN in the limbic basal ganglia circuits (Le Jeune et al., 2010). The results of their study would not only help researchers to explain emotional modifications following STN DBS in PD, but would also provide information for operational hypotheses about the non-motor functional role of the STN.

The limbic and associate circuits modulate and integrate neuronal processes that are related to patients’ emotion. The limbic neurocircuit includes the anterior cingulate cortex and OFC (Temel et al., 2005). It is crucial for identifying the emotional significance of stimuli and for generating an effective response to these stimuli. The brain region in our study related to cognitive neurocircuit includes the OFC. The inferior frontal gyrus was reported to be functionally connected to the OFC (Rolls et al., 2020). The high level cognitive processes associated with the basal ganglia are thought to depend on connections between the frontal, temporal, parietal, and striatum. Metabolic changes in these regions participated in the network and integrate emotion relayed through the STN, the stimulation to STN may resulting in emotion regulation.

This study adds to the evidence that STN-DBS can alleviate depressive symptoms in patients with PD (Funkiewiez et al., 2004; Chopra et al., 2014; Birchall et al., 2017). The present results extend previous studies by indicating that the antidepressant action of STN-DBS is associated with the restoration of glucose metabolic change in brain regions critically involved in emotion regulation. Although it has occasionally been reported that depressive symptoms worsened after STN-DBS treatment, such unintended effects probably stem from a fairly abrupt, substantial reduction of dopaminergic medication usage after the initiation of STN-DBS (Voon et al., 2006).

To the best of our knowledge, this study is the first that has detected significant correlations between improvements in depressive symptoms and restoration of brain glucose metabolism in patients with PD following STN-DBS treatment.

Study found that depression patients showed greater glucose uptake in ventrolateral prefrontal cortical and paralimbic regions compared to healthy subjects (Dunn et al., 2002). Another study demonstrate that depression and chronic stress exposure can lead to atrophy of neurons in cortical and limbic brain regions implicated in depression (Duman et al., 2019). ALE analyses showed the brain metabolism in bilateral insula, left lentiform nucleus putamen, right caudate and cingulate gyrus were significantly decreased. The brain activity in right thalamus pulvinar and declive of posterior lobe were significantly increased in MDD patients (Su et al., 2014). However, these studies had inconsistent results.

Repeat PET imaging was conducted in several cohorts of PD patients (Patients with depression, anxiety, and dementia were excluded). There was characterized by increases in pallidothalamic, pontine, and cerebellar metabolic activity associated with relative reductions in the lateral premotor cortex, supplementary motor area, and posterior association cortices (Ma et al., 2007). There are significant differences in cerebral glucose metabolism between PD patients and depression patients. The abnormal brain regions of patients with depression are mainly concentrated in the related regions of the limbic system, while in PD patients, mostly located in motor circuit and cerebellum (Temel et al., 2005).

For the comparison of brain metabolism and motor scores between PD patients and PD patients with depression, the brain metabolism of PD patients without depression has typical characteristics and is stable, which were highly reproducible. The research of Yilong Ma and Chunyan Cao pointed out that the above metabolic patterns will have characteristic and repeatable changes after STN-DBS (Ma et al., 2007; Cao et al., 2017). In our study, the brain regions related to metabolic changes are right inferior frontal gyrus, right OFC and right lingual gyrus, these areas are closely related to limbic circuit (Temel et al., 2005; Germain et al., 2007; Rolls et al., 2020). The parietal cortex is connected with the OFC (Kahnt et al., 2012). It is different from the metabolic characteristics of PD patients without depression.

In terms of the impact of surgery on patients’ motor function, As previously reported (Birchall et al., 2017), depressed PD patients who underwent unilateral STN DBS had a significant improvement in motor symptoms, measured by the UPDRS-III in the off-medication state, at 3 months post-operatively (20.95 ± 1.42) compared to pre-operative baseline (35.16 ± 1.39). Stimulation induced a significant improvement of 40% (*P* < 0.01) in the off-medication UPDRS-III score. In addition (Antosik-Wójcińska et al., 2017), after bilateral STN-DBS, depression in patients with Parkinson’s depression improved by 21%, and UPDRS improved by 51% compared with baseline (*P* < 0.01).

Consistently, in our study at 1-year follow-up, the severity of patients’ motor symptoms was improved by 54.0 ± 5.6% compared with baseline, measured by the UPDRS-III in the off-medication state. Both our study and the previous one found there was no correlation between the improvement of UPDRS and HAMD.

Several study limitations should be acknowledged. First, the study was an observational study, so that bias and confounding cannot be excluded. Accordingly, the results should be interpreted with caution. Second, the study may have suffered from an attrition bias since not all patients initially enrolled in the study underwent PET imaging at 1-year follow-up. However, the latter possibility is unlikely because patients who either did or did not undergo follow-up PET imaging were not significantly different from each other in demographic and clinical characteristics at baseline and clinical outcome at 1-year follow-up. Third, the study included a relatively small number of patients and healthy controls, which may have reduced the statistical power to detect certain effects. Finally, the duration of follow-up was relatively short. Therefore, it remains to be determined whether the antidepressant action of STN-DBS in patients with PD persists over the long-term course of the treatment and illness.

## Data Availability Statement

The original contributions presented in the study are included in the article/Supplementary Material, further inquiries can be directed to the corresponding authors.

## Ethics Statement

The studies involving human participants were reviewed and approved by Ethics Committee of Ruijin Hospital, Shanghai Jiao Tong University School of Medicine. The patients/participants provided their written informed consent to participate in this study. Written informed consent was obtained from the individual(s) for the publication of any potentially identifiable images or data included in this article.

## Author Contributions

XZ and CZ designed and conceptualized the study, and drafted the manuscript for intellectual content. HZ contributed to acquisition of data, analyzed the data, and drafted the manuscript for intellectual content. ZL analyzed and interpreted the data, and revised the manuscript for intellectual content. DB, YL, CH, and VV revised the manuscript for intellectual content. DL designed and conceptualized the study, interpreted the data, and revised the manuscript for intellectual content. BS designed and conceptualized the study. All authors contributed to the article and approved the submitted version.

## Conflict of Interest

The authors declare that the research was conducted in the absence of any commercial or financial relationships that could be construed as a potential conflict of interest.

## Publisher’s Note

All claims expressed in this article are solely those of the authors and do not necessarily represent those of their affiliated organizations, or those of the publisher, the editors and the reviewers. Any product that may be evaluated in this article, or claim that may be made by its manufacturer, is not guaranteed or endorsed by the publisher.
