# Supplementary material for: Effects of Bilateral Subthalamic Nucleus Stimulation on Depressive Symptoms and Cerebral Glucose Metabolism in Parkinson’s Disease: A 18F-Fluorodeoxyglucose Positron Emission Tomography/Computerized Tomography Study
Source: Front Neurosci. 2022 Jun 1;16:843667. doi: 10.3389/fnins.2022.843667 (PMC9200334; doi:10.3389/fnins.2022.843667)
Supplement: Supplementary file 1 [file Table_1.docx]

**Table S1. Regions with modified glucose hypermetabolism and hypometabolism in PD-TRD patients compared to these regions in healthy individuals.**

| **Brain regions** | **Side** | **Coordinates^a^** | | | **BA** | **Zmax** |
| --- | --- | --- | --- | --- | --- | --- |
|  |  | **X** | **Y** | **Z** |  |  |
| *Metabolic increase^b^* |  |  |  |  |  |  |
| Lentiform nucleus | Bilateral | 14 | -8 | -4 | * | 4.59 |
| Claustrum | Bilateral | -28 | 12 | -8 | * | 4.67 |
| Subcallosal gyrus | Right | 18 | 14 | -12 | 13 | 4.65 |
| Parahippocampal gyrus | Bilateral | 40 | -24 | -16 | 36 | 4.43 |
| Medial frontal gyrus | Bilateral | 22 | 48 | 16 | 10 | 4.53 |
| Inferior frontal gyrus | Bilateral | 52 | 42 | 4 | 46 | 4.91 |
| Superior frontal gyrus | Bilateral | 28 | 56 | -4 | 10 | 4.83 |
| Anterior cingulate | Bilateral | 22 | 42 | 4 | 32 | 4.01 |
| Orbital gyrus | Right | 14 | 50 | -30 | 11 | 3.83 |
| Precentral gyrus | Bilateral | -58 | 0 | 10 | 6 | 3.61 |
| Insula | Right | 42 | 2 | 20 | 13 | 3.24 |
| Superior temporal gyrus | Right | 50 | 18 | -40 | 38 | 3.32 |
| Middle temporal gyrus | Right | 64 | 6 | -14 | 21 | 3.58 |
| Amygdala | Right | 34 | -4 | -22 | * | 4.72 |
| Cerebellar tonsil | Left | -30 | -54 | -50 | * | 5.31 |
| *Metabolic decrease^b^* |  |  |  |  |  |  |
| Lingual gyrus | Bilateral | 4 | -88 | -4 | 18 | 5.4 |
| Cuneus | Bilateral | -2 | -76 | 8 | 23 | 5.23 |
| Superior parietal lobule | Bilateral | 28 | -64 | 56 | 7 | 5.09 |
| Precuneus | Bilateral | -18 | -82 | 42 | 7 | 4.9 |
| Middle occipital gyrus | Bilateral | 22 | -98 | 12 | 19 | 4.46 |
| Inferior parietal lobule | Bilateral | -48 | -48 | 54 | 40 | 4.03 |
| Postcentral gyrus | Left | -42 | -32 | 68 | 1 | 3.82 |
| Angular gyrus | Bilateral | -36 | -80 | 30 | 39 | 3.64 |
| Inferior occipital gyrus | Right | 40 | -84 | -16 | 18 | 3.43 |
| Middle temporal gyrus | Left | -32 | -62 | 16 | 21 | 3.31 |
| Middle frontal gyrus | Left | -30 | 6 | 68 | 6 | 3.48 |

Regions with modified glucose hypermetabolism and hypometabolism in PD-TRD patients, compared to these regions in healthy individuals. (p < 0.01, uncorrected; extend threshold ≤ 100).

^a^ The Montreal Neurological Institute (MNI) standard brain space.

^b^ Regions that survived false discovery rate (FDR) correction (p < 0.05).

Abbreviation: BA = Brodmann’s area; PD-TRD = Parkinson’s disease with treatment-resistant depression
